# Treatment of a Complex Personality Disorder Using Repeated Doses of LSD—A Case Report on Significant Improvements in the Absence of Acute Drug Effects

**DOI:** 10.3389/fpsyt.2020.573953

**Published:** 2020-10-22

**Authors:** Felix Müller, Markus Mühlhauser, Friederike Holze, Undine E. Lang, Marc Walter, Matthias E. Liechti, Stefan Borgwardt

**Affiliations:** ^1^University of Basel, Department of Psychiatry (Universitäre Psychiatrische Kliniken), Basel, Switzerland; ^2^University of Basel, Division of Clinical Pharmacology and Toxicology, Department of Biomedicine and Department of Clinical Research, Basel, Switzerland; ^3^Department of Psychiatry and Psychotherapy, University of Lübeck, Lübeck, Germany

**Keywords:** LSD, treatment, depression, personality disorder, hallucinogen, case report

## Abstract

A 39-year-old female patient suffering from severe, treatment-resistant depression and other symptoms associated with a complex personality disorder was admitted to our open psychiatric ward for an experimental treatment with lysergic acid diethylamide (LSD). The substance was administered in repeated weekly and ascending doses. Curiously, there were no substantial acute subjective effects of the drug despite adequate dosing, which was also confirmed by plasma drug concentration monitoring. However, the patient showed rapid and significant improvement with most notable changes in depressed mood, emotional instability, loss of energy, and suicidal ideations. Additionally, the SCL-90 questionnaire indicated significant decreases in global severity and in various psychopathological subscales. Improvements persisted for ~7 days after each administration. Due to the severe course of the illness and the resistance to previous treatment it was decided to continue this experimental approach with weekly repeated doses of LSD. The patient will be observed closely with regard to somatic and mental side effects. Two features of this case are remarkable: Firstly, administration of LSD was associated with significant improvements in various symptoms of a condition usually difficult to treat. Secondly, symptom reductions occurred in the absence of acute drug effects. Therefore, the mechanism of action seemed to deviate from the concept that improvements after administration of drugs like LSD are due to experiences during the acute drug effects. This case might indicate that LSD can induce rapid but transient beneficial effects on several psychopathological symptoms. The time course of these improvements resembled antidepressant effects seen after administration of ketamine.

## Background

Several studies have indicated that limited administrations of hallucinogenic drugs can induce very long-lasting improvements in anxiety and depressive symptoms [e.g., (1, 2)]. Similar results were found for the psychoactive substance MDMA (3). Commonly, therapeutic effects of these substances are seen as resulting from subjective experiences during the acute effects of the respective drug and are described as outlasting the pharmacological action by far (4). In this article, we report on the case of a patient suffering from treatment-resistant depression associated with combined personality disorder who was referred to an experimental treatment using this approach.

## Case Presentation

A 39-year-old female patient was referred to our psychiatric hospital because of a depressive syndrome which had been present for months and had worsened over time. On admission, the patient mainly complained about depressed mood, anhedonia, loss of energy, fatigue, feelings of worthlessness, and suicidal ideations. The psychiatric medication at the time of admission consisted in daily doses of 300 mg bupropion, 337.5 mg venlafaxine, 600 mg pregabalin, 1.5 mg risperidone, and 7.5 mg lorazepam. She also received regular nasal administration of racemic ketamine, most recently 112 mg two times a week. According to the patient, ketamine slightly diminished symptoms for ∽24 h. She also reported that pregabalin and risperidone were helpful for feeling “more calm” while she didn't report beneficial effects with regard to the antidepressant medication.

Regarding her history, the patient reported that first symptoms were present as a teenager, where she had felt lonely and detached, had suffered from anhedonia, feelings of worthlessness, depressed mood, and suicidal ideations. She excessively consumed alcohol and cannabis. Later in life, visual and tactile pseudohallucinations occurred (particularly the feeling of being touched by someone and seeing snakes on the floor). She also developed panic attacks and compulsive thoughts and acts which were mostly related to fears of contamination. For the first time she sought treatment at the age of 22 after her partner had committed suicide. Subsequently, she received psychotherapeutic and psychiatric treatment for 3 years. The patient was admitted to our hospital for the first time aged 30 after a suicide attempt by intoxication with several drugs. Directly after this hospitalization, she attended a regular psychodynamic psychotherapy which was still ongoing at the time of admission. Over the last years, the patient had been treated with several psychiatric drugs, including antidepressants of different types (escitalopram, sertraline, fluoxetine, duloxetine, moclobemide, reboxetine, trazodone, mirtazapine, vortioxetine, nortriptyline), mood stabilizers (lithium, lamotrigine, valproate), antipsychotics (aripiprazole, quetiapine, olanzapine), and stimulants (modafinil, methylphenidate, atomoxetine). She also had been using benzodiazepines on a regular basis, mostly to cope with fears of contamination.

The patient was married and mother of an infant. She didn't graduate from school but worked as a common laborer. Later on, she qualified for a disability pension due to her mental condition.

On admission, the psychopathology appeared to be complex. Among others, previous diagnoses included depression, bipolar disorder, Asperger syndrome, attention deficit syndrome, personality disorders, agoraphobia, and panic attacks.

During treatment in our hospital, the patient was assessed using SCID-I and SCID-II. According to the criteria of the ICD-10 classification, the patient was diagnosed with a mixed personality disorder with emotionally unstable, paranoid, and compulsive features (F61.0). Furthermore, the SCID assessment indicated that the criteria for severe, recurrent depression (F33.2) and for mixed obsessional thoughts and acts (F42.2) were met. Mostly, the clinical presentation was dominated by severe depressive symptoms including chronic suicidal thoughts and features of the personality disorder. The clinical course of the previous years had been severe and hard to influence.

## Treatment

Considering the severity of the disease and the insufficient efficacy of previous treatments, it was decided to request special permission for an experimental treatment using the psychoactive drugs MDMA and LSD which can be granted by the Swiss authorities for specific patients. This decision was not taken lightly. Some features of the patient's psychopathology can be regarded as close to psychosis and especially hallucinogenic drugs like LSD are potentially harmful in such cases (4). Therefore, our evaluation of risks and potential benefits was made carefully. This comprised the severe course of the disease, the clinical presentation with special regard to psychotic symptoms and the risk profile of the substances MDMA and LSD. It was also taken into account, that previous evidence indicated that hallucinogens can be administered safely in emotional unstable personality disorders under controlled conditions (5). It was also noted that regular administrations of different psychostimulants and ketamine had never resulted in psychosis-like symptoms in this patient.

**Figure 1:**
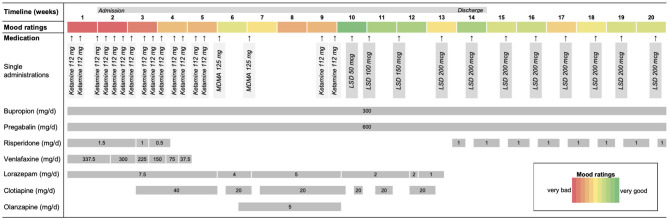
Time course of the treatment including concomitant psychiatric medications. Venlafaxine and olanzapine were tapered off in order to avoid possible pharmacological interactions. Risperidone and clotiapine were paused prior each administration for the same reason. Lorazepam was tapered off for clinical reasons. The figure also shows the patient's mood ratings for each week.

Prior to this experimental treatment, venlafaxine and olanzapine were tapered off to avoid pharmacological interactions with MDMA and LSD, as these substances mainly act at the serotonin system (4). Risperidone and clotiapine were paused prior to each administration for the same reason. Lorazepam was tapered off for clinical reasons. Figure 1 gives an overview of the whole course of the treatment, including all psychiatric medications.

Treatment was then started with two single oral administrations of 125 mg MDMA. This was based on the possibility of therapeutic effects (3), but was mainly intended to accustom the patient to drug-induced mental alterations by using a substance with a moderate profile of effects (6). MDMA was administered in a setting similarly used in several studies on therapeutic effects of MDMA and hallucinogens [e.g., (1, 2, 7)]. Sessions were started in the morning and the patient stayed in a quiet room during the acute drug effects. Blood pressure and temperature were monitored regularly. Some music was played and guidance and support was offered by a therapist who was experienced in conducting such sessions. Later, the same setting was used for the administrations of LSD.

Although symptoms improved temporarily, the patient relapsed relatively quickly. Treatment with LSD was then initiated with an oral administration of 50 μg dose. There was an interval of 3 weeks between the last exposure to MDMA and the first administration of LSD which allowed full washout of MDMA [half-life ~8 h; (8, 9)] and recovery of the serotonin system (10). The low dose of 50 μg was chosen for safety reasons, but also to carefully accustom the patient to the effects of the drug. This was based on our experiences in a dose-finding study in healthy participants where 50 μg induced subtle, but typical drug effects (11). The patient reported no typical drug effects at this dose, but noted elevated mood ∽1.5 h after intake. Subsequently, the patient received weekly administrations of LSD and the dose was gradually increased to 100, 150, and 200 μg, respectively. Based on our experience, substantial effects were expected at doses ~100 μg (11, 12). However, the patient reported only minor acute effects after these doses, stating that she felt “a bit like being slightly drunk” for a few hours. Notably, the patient reported that her mood and loss of energy improved considerably after each intake. This was also reflected by the patient's mood diary, which is shown in Figure 1. Moreover, she also stated that she felt “more calm and stable” and that her suicidal thoughts had diminished.

These changes were also observed by the attending physicians and the ward staff. After a few administrations, additional decreases in compulsive thoughts and acts were observed. It was also noticed, that the improvement seemed to be dose-dependent. Overall, the condition improved significantly during these weeks. No relevant side effects occurred during the whole treatment.

**Figure 2:**
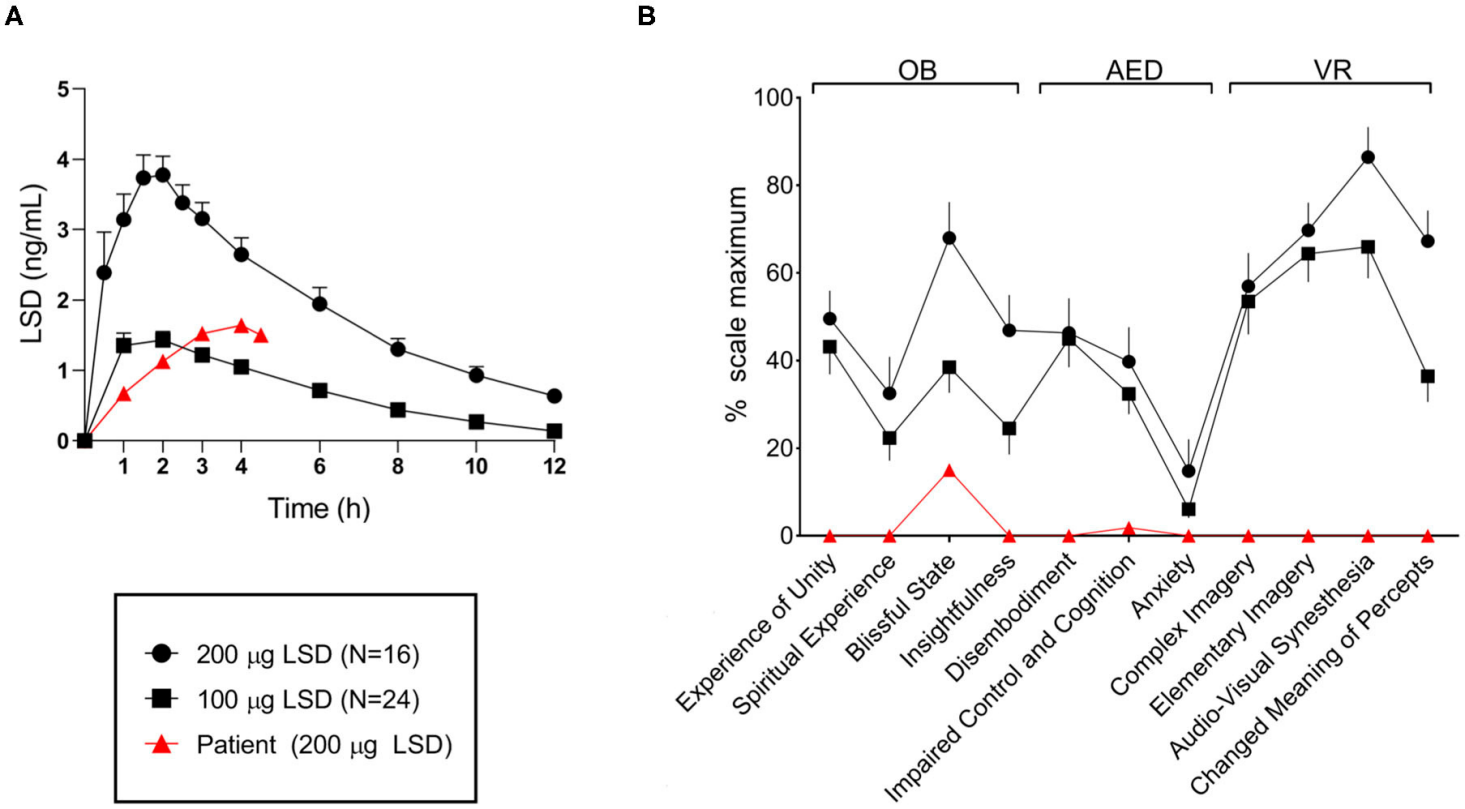
**(A)** Patient's plasma LSD concentration-time curve after administration of 200 μg up to 4.5 h compared with concentrations in a sample of healthy subjects after 100 and 200 μg LSD, respectively. The patient's plasma concentration roughly corresponded to those seen after 100 μg and the time of peak concentration was delayed. **(B)** Patient's acute drug effects on the questionnaire 5D-ASC after administration of 200 μg compared with a sample of healthy subjects after 100 and 200 μg LSD, respectively. Only some effects were observed on the scales “blissful state” and “impaired control and cognition” and those effects were substantially lower compared with both samples. Figures were adopted from: (12). All data is expressed as mean and standard error of the mean.

In order to inquire the absence of the expected acute effects, plasma levels were taken hourly after administration of the 200 μg dose. Plasma samples were analyzed using a validated mass spectrometry method (13). Plasma concentrations were slightly lower than expected and the time of peak was delayed (please see Figure 2A). However, these findings did not explain why acute effects were almost absent. Additionally, acute subjective effects of the drug were quantified using a standard questionnaire for hallucinogenic drugs (5D-ASC: 5 Dimensions of Altered States of Consciousness Questionnaire). Some effects were seen on the dimensions “blissful state” and “impaired control and cognition.” However, these effects were considerably less pronounced than expected on the basis of the dose and the observed plasma concentrations. All other, more “typical” drug effects (like visual alterations) were completely missing which confirmed the clinical impression (please see Figure 2B for more details).

**Figure 3:**
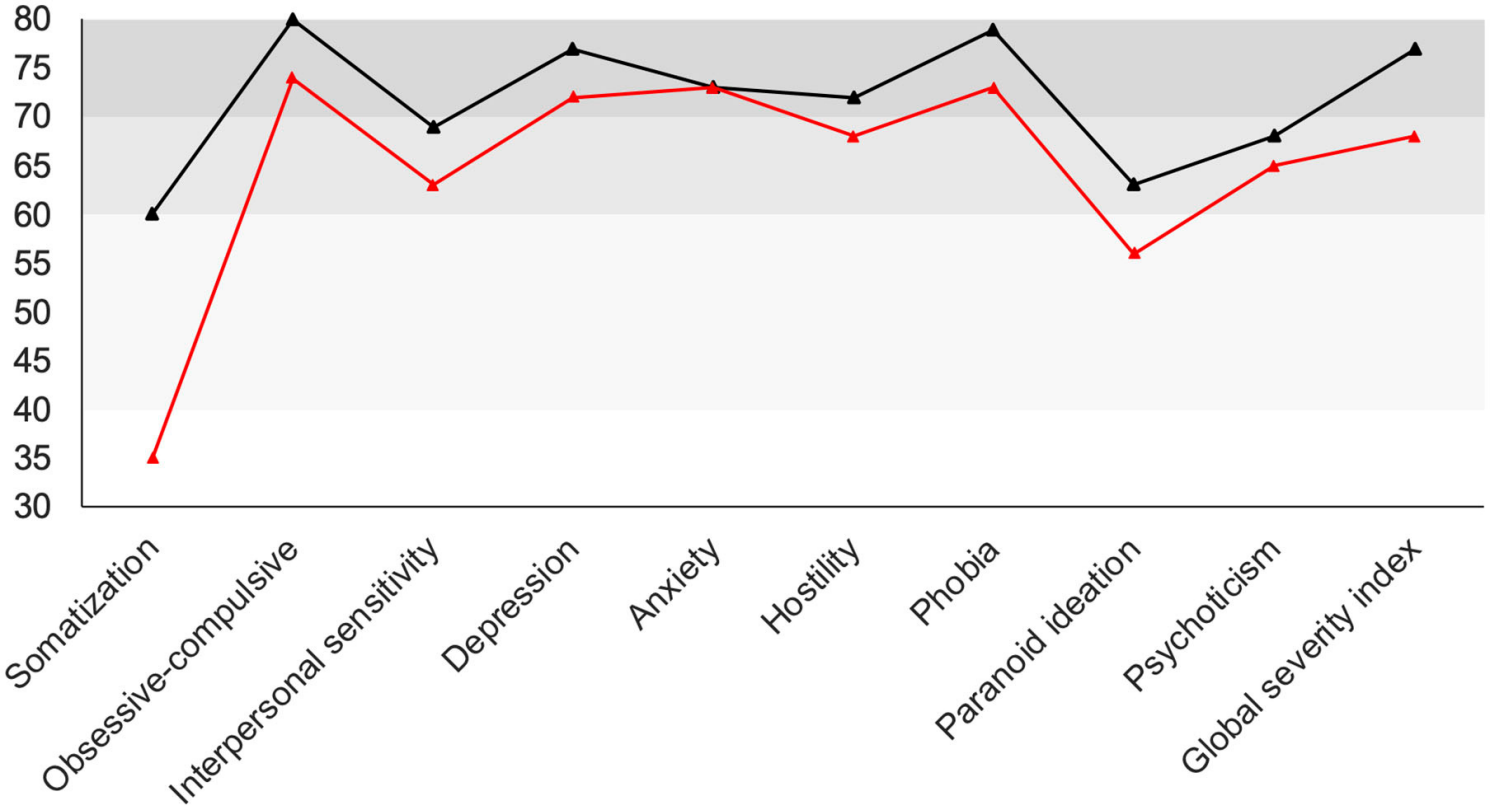
SCL-90 scales before (black line) and after (red line) treatment. Scores of the patient are normalized for gender and age (t-transformation). Gray areas indicate the “normal” (40–60), “clinically suspicious” (60–70), and “clinically relevant” (70–80) range. Significant decreases were observed in the scales “somatization,” “obsessive-compulsive,” “interpersonal sensitivity,” “depression,” “hostility,” “phobia,” “paranoid ideation,” and the “global severity index”.

Subsequently, the patient received weekly administrations of 200 μg LSD. The overall condition remained stable and improvements were considered to be sufficient for discharge. Directly before leaving our inpatient service, the patient completed another version of the questionnaire SCL-90 (Symptom Checklist-90), which she also had completed prior to this treatment. The SCL-90 covers a broad spectrum of symptoms and can be used for the assessment of therapeutic effects. According to the reliable change index (14), significant decreases were observed in the scales “somatization,” “obsessive-compulsive,” “interpersonal sensitivity,” “depression,” “hostility,” and “phobia.” The global severity index was also significantly decreased. No significant alterations were observed for “anxiety,” “paranoid ideation,” and “psychoticism” (Figure 3).

As described above, the course of the disease had been severe up to this point and the patient did not respond to several therapeutic approaches. Considering this history, it was decided to continue the treatment with weekly administrations of 200 μg LSD after discharge. In our outpatient treatment, improvement persisted. It was also noted, that the patient did not use benzodiazepines again, which she did for several years at least 3 days a week (due to specific fears of contamination during these days). Again, this decision was not taken lightly. LSD is generally regarded as non-addictive and physically non-toxic but this assessment is mostly based on experiences with sporadic use (4). Therefore, the risk profile of frequent administrations is unknown. A decrease of the administered dose and/or an extension of the intervals between administrations is currently planned.

The patient will be observed closely with regard to somatic and mental side effects and treatment will be stopped in case of suspected adverse events. This will comprise cognitive testing, laboratory, and a general physical examination. More specifically, the patient will be screened for valvular heart disease (using electrocardiogram and echocardiography), a disease which has been associated with long-term intake of agonists at the serotonin_2B_-receptor (15).

## Discussion

The response of the patient was surprising and we can only speculate with regard to the underlying mechanism. Overall, the case resembles some features which have been described for “microdosing” of psychedelics, e.g., the repeated use of small doses of serotonergic hallucinogens in order to transiently improve mood, energy and other domains (16). It is believed that small doses below the threshold for “typical” hallucinogenic effects still can induce relevant stimulation of the serotonin_2A_-receptor, resulting in positive mental effects which last for a few days (16). Somehow, this resembles our case, where only minor acute effects, but transient improvements in several domains were observed. However, the doses in this patient were considerably higher than those described for “microdosing” (where doses of 10 μg LSD are typically used). The absence of acute drug effects in our patient might be explained by specific physiological alterations like a serotonin_2A_-receptor polymorphism which might influence the action of the drug. It is also possible, that regular intake of psychopharmacological medication indirectly interfered with LSD, e.g., GABAergic effects of pregabalin might prevent unfolding of specific hallucinogenic effects.

However, the observed improvements might be due to factors which are not related to any specific pharmacological mechanism. It is conceivable, that the response was due to placebo effect, which might have been exaggerated by the commonly assumed “potency” of this substance which might also suggest therapeutic efficacy. On the other side, the long history of psychopharmacological treatments does not suggest that the patient is particularly prone to these effects. Moreover, the patient was primed according to the concept of “hallucinogenic” therapy. This comprised descriptions of the expected effects and the model for therapeutic effects associated with this form of treatment. The possibility of improvements in the absence of acute drug effects was not mentioned. It is also possible, that the patient benefitted from features of the laborious procedure, including, for example, many hours of monitoring. However, the patient's response to the treatment with MDMA (which comprised the same procedures) was quite different and we didn't have the impression that less time consuming procedures at the end of treatment (i.e., the patient was only monitored very briefly) had any such effect.

Overall, this case offers two interesting features: Firstly, administration of LSD was associated with improvements in a broad spectrum of psychopathological symptoms in a condition that was very difficult to treat. Secondly, these improvements occurred in the absence of distinct hallucinogenic drug effects. This deviates from the concept that improvements after administration of drugs like LSD and MDMA are due to specific peak or mystical experiences during the acute drug effects. Another deviation with regard to this concept was that symptom reductions diminished after ∽1 week while studies report very long-lasting improvements [e.g., (1, 3)]. These features might indicate that LSD can induce effects resembling those after administration of ketamine, which induces rapid but transient antidepressant effects.

## Data Availability Statement

All datasets presented in this study are included in the article/supplementary material.

## Ethics Statement

Written informed consent was obtained from the individual(s) for the publication of any potentially identifiable images or data included in this article.

## Author Contributions

FM conducted the treatment with MDMA and LSD, collected the data, and wrote the initial draft of the manuscript. MM provided regular psychiatric treatment. FH and ML analyzed plasma drug concentrations. MM, UL, MW, ML, and SB provided expertise and advice. All authors contributed to the article and approved the submitted version.

## Conflict of Interest

The authors declare that the research was conducted in the absence of any commercial or financial relationships that could be construed as a potential conflict of interest.

## References

[1] Gasser P Kirchner K Passie T. LSD-assisted psychotherapy for anxiety associated with a life-threatening disease: a qualitative study of acute and sustained subjective effects. J Psychopharmacol. (2015) 29:57–68. 10.1177/0269881114555249 25389218

[2] Griffiths RR Johnson MW Carducci MA Umbricht A Richards WA Richards BD. Psilocybin produces substantial and sustained decreases in depression and anxiety in patients with life-threatening cancer: a randomized double-blind trial. J Psychopharmacol. (2016) 30:1181–97. 10.1177/0269881116675513 27909165 PMC5367557

[3] Mithoefer MC Grob CS Brewerton TD. Novel psychopharmacological therapies for psychiatric disorders: psilocybin and MDMA. Lancet Psychiatry. (2016) 3:481–8. 10.1016/S2215-0366(15)00576-3 27067625

[4] Nichols DE. Psychedelics. Pharmacol Rev. (2016) 68:264–355. 10.1124/pr.115.011478 26841800 PMC4813425

[5] Zeifman RJ Wagner AC Exploring the case for research on incorporating psychedelics within interventions for borderline personality disorder. J Context Behav Sci. (2020) 15:1–11. 10.1016/j.jcbs.2019.11.001

[6] Holze F Vizeli P Müller F Ley L Duerig R Varghese N. Distinct acute effects of LSD, MDMA, and d-amphetamine in healthy subjects. Neuropsychopharmacology. (2020) 45:462–71. 10.1038/s41386-019-0569-3 31733631 PMC6969135

[7] Mithoefer MC Mithoefer AT Feduccia AA Jerome L Wagner M Wymer J. 3,4-methylenedioxymethamphetamine (MDMA)-assisted psychotherapy for post-traumatic stress disorder in military veterans, firefighters, and police officers: a randomised, double-blind, dose-response, phase 2 clinical trial. Lancet Psychiatry. (2018) 5:486–97. 10.1016/S2215-0366(18)30135-4 29728331

[8] Hysek CM Simmler LD Ineichen M Grouzmann E Hoener MC Brenneisen R. The norepinephrine transporter inhibitor reboxetine reduces stimulant effects of MDMA (ecstasy) in humans. Clin Pharmacol Ther. (2011) 90:246–55. 10.1038/clpt.2011.78 21677639

[9] Kolbrich EA Goodwin RS Gorelick DA Hayes RJ Stein EA Huestis MA. Plasma pharmacokinetics of 3,4-methylenedioxymethamphetamine after controlled oral administration to young adults. Ther Drug Monit. (2008) 30:320–32. 10.1097/FTD.0b013e3181684fa0 18520604 PMC2663855

[10] Scheffel U Lever JR Stathis M Ricaurte GA. Repeated administration of MDMA causes transient down-regulation of serotonin 5-HT2 receptors. Neuropharmacology. (1992) 31:881–93. 10.1016/0028-3908(92)90126-A 1359444

[11] Holze F Vizeli P Laura L Müller F Dolder P Stocker M Acute dose-dependent effects of LSD in a double-blind placebo-controlled study in healthy subjects. Neuropsychopharmacology. (2020). 10.1038/s41386-020-00883-6 PMC8027607 33059356

[12] Liechti ME Dolder PC Schmid Y. Alterations of consciousness and mystical-type experiences after acute LSD in humans. Psychopharmacology. (2017) 234:1499–510. 10.1007/s00213-016-4453-0 27714429 PMC5420386

[13] Dolder PC Liechti ME Rentsch KM. Development and validation of a rapid turboflow LC-MS/MS method for the quantification of LSD and 2-oxo-3-hydroxy LSD in serum and urine samples of emergency toxicological cases. Anal Bioanal Chem. (2015) 407:1577–84. 10.1007/s00216-014-8388-1 25542574

[14] Schauenburg H Strack M. Measuring psychotherapeutic change with the symptom checklist SCL 90 R. Psychother Psychosom. (1999) 68:199–206. 10.1159/000012333 10396011

[15] Hutcheson JD Setola V Roth BL Merryman WD. Serotonin receptors and heart valve disease-It was meant 2B. Pharmacol Ther. (2011) 132:146–57. 10.1016/j.pharmthera.2011.03.008 21440001 PMC3179857

[16] Kuypers KPC Ng L Erritzoe D Knudsen GM Nichols CD Nichols DE. Microdosing psychedelics: more questions than answers? An overview suggestions for future research. J Psychopharmacol. (2019) 33:1039–57. 10.1177/0269881119857204 31303095 PMC6732823

